# Pyridine Scaffolds, Phenols and Derivatives of Azo Moiety: Current Therapeutic Perspectives

**DOI:** 10.3390/molecules26164872

**Published:** 2021-08-11

**Authors:** Tehreem Tahir, Muhammad Ashfaq, Muhammad Saleem, Muhammad Rafiq, Mirza Imran Shahzad, Katarzyna Kotwica-Mojzych, Mariusz Mojzych

**Affiliations:** 1 Faculty of Science, Institute of Biochemistry, Biotechnology & Bioinformatics, The Islamia University of Bahawalpur, Bahawalpur 63100, Pakistan; tahreemtahir90@gmail.com; 2 Faculty of Science, Institute of Chemistry, The Islamia University of Bahawalpur, Bahawalpur 63100, Pakistan; chashfaqiub@yahoo.com; 3 Department of Chemistry, Sub-Campus Bhakkar, University of Sargodha, Bhakkar 30000, Pakistan; saleemqau@gmail.com; 4 Department of Physiology and Biochemistry, Faculty of Bio-Sciences, Cholistan University of Veterinary and Animal Sciences, Bahawalpur 63100, Pakistan; mrafiq@cuvas.edu.pk; 5 Department of Histology, Embryology and Cytophysiology, Medical University of Lublin, Radziwiłłowska 11, 20-080 Lublin, Poland; katarzynakotwicamojzych@umlub.pl; 6 Department of Chemistry, Siedlce University of Natural Sciences and Humanities, 3-Maja 54, 08-110 Siedlce, Poland

**Keywords:** azo, heterocyclic, hydroxyl, therapeutic, phenol, pyridine

## Abstract

Synthetic heterocyclic compounds have incredible potential against different diseases; pyridines, phenolic compounds and the derivatives of azo moiety have shown excellent antimicrobial, antiviral, antidiabetic, anti-melanogenic, anti-ulcer, anticancer, anti-mycobacterial, anti-inflammatory, DNA binding and chemosensing activities. In the present review, the above-mentioned activities of the nitrogen-containing heterocyclic compounds (pyridines), hydroxyl (phenols) and azo derivatives are discussed with reference to the minimum inhibitory concentration and structure–activity relationship, which clearly indicate that the presence of nitrogen in the phenyl ring; in addition, the hydroxyl substituent and the incorporation of a diazo group is crucial for the improved efficacies of the compounds in probing different diseases. The comparison was made with the reported drugs and new synthetic derivatives that showed recent therapeutic perspectives made in the last five years.

## 1. Introduction

Nitrogen-, oxygen- and sulfur-based heterocyclic compounds are contributing extensively in the current medicinal chemistry because of their wide applications in drug discovery and development [1,2,3,4,5,6,7,8]. As the potentially privileged scaffolds, pyridine-fused heterocycles have attracted attention due to their effective and widespread biological properties for the treatment of different diseases such as cancer, diabetes, hyperpigmentation, tuberculosis, hyperplasia and ulcer [9,10,11,12,13,14,15,16]. 

The hydroxyl groups found in the phenolic compounds have been granted a vital place in medicinal chemistry because they are responsible for anti-oxidant, antiviral, antimicrobial, insecticidal, anti-sclerotic, anti-parasitic and hypoglycemic biological activities, among others. The utilization of these derivatives in organic synthesis has become a traditional approach for the preparation of wide range of phenol derivatives [17,18].

Azo dyes are one of the most significant classes of chromophores with diverse applications in science and industry, with simplistic synthesis approach can yield a broad spectrum derivatives. Specifically, the synthesis of heterocyclic azo dyes has gained particular attention from the several decades as they exhibit antifungal, anti-convulsant, anti-inflammatory, anti-tubercular, DNA binding and analgesic properties [19,20,21,22,23,24,25,26,27,28].

The present review is devoted to therapeutic potential of pyridine scaffolds, phenols and derivatives of azo moiety in the field of medicinal chemistry. In this review, the pharmacological potential of the mentioned compounds have been discussed separately with structure–activity relationship, and the conclusion is drawn to co-relate the synergistic effect of pyridine rings fused with azo-phenol derivatives in the systematic manner. 

### Role of Pyridine Scaffolds in Medicinal Chemistry

Pyridine moieties are naturally present in many molecules such as vitamins, co-enzymes and alkaloids. Pyridine scaffolds are known for antimicrobial, antiviral, antioxidant, anti-inflammatory, antiamoebic, antidiabetic, antimalarial and anti-psychotic medicinal properties, hence are a part of many drugs and found to enhance pharmacological characteristics of the drugs because of their weak basicity and aqueous solubility [29]. In this aspect, a novel series of pyrimidine–pyridine hybrids was synthesized by combining pyridine moieties with pyrimidine scaffolds (**1a**). The anti-inflammatory activity of the hybrid molecules was evaluated and it was found after the detailed structure–activity relationship that a derivative having a trimethoxy phenyl group at position 4 of the pyridine ring was the most active candidate with edema inhibitory percent = 74% after 1 h. Hence, it was concluded that by increasing the number of methoxy groups on the phenyl ring directly attached to the pyridine scaffold enhanced the anti-inflammatory activity of the whole ring system [30]. Similarly, a new series of pyrrolo[2,3-*b*]pyridine derivatives was evaluated against human neutrophil elastase (HNE) inhibitors with some modifications such as; shift of nitrogen atom from position 2 to 7 in previously synthesized indazole ring. After that, a variety of different substitutions were done at different positions, e.g., insertion of alkyl carbonyl, methylbenzoyl and removal of cyanide group was found to be favorable in HNE inhibitory activity. It was also confirmed that the presence of 1-*N*-CO and other electron withdrawing groups was necessary for the potent inhibitory activity against HNE (**1b**) [31]. 

**Figure 1:**
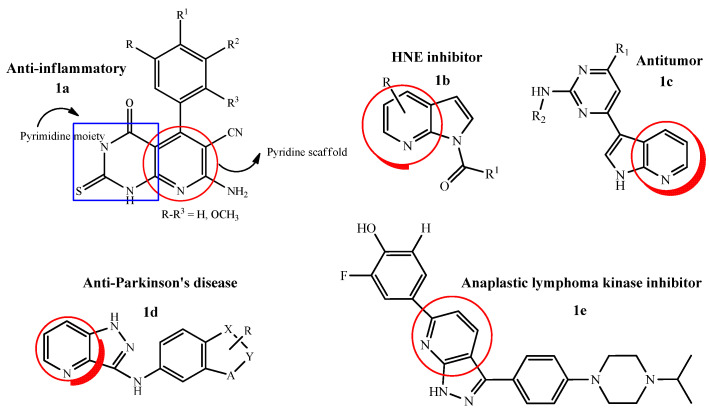
Biologically active pyrrolopyridine hybrid molecules.

New classes of 3-substituted 1*H*-pyrrolo[2,3-*b*]pyridine analogues were designed and evaluated for their in vitro anti-tumor activity against maternal embryonic leucine zipper kinase (MELK). Among all the tested title compounds, one 1*H*-pyrrolo[2,3-*b*]pyridine derivative (**1c**) exhibited excellent anti-MELK potential (IC_50_ 32 nM) and anti-proliferative activity (IC_50_ range 0.109 µM to 0.245 µM) against A549, MDA-MB-231 and MCF-7 cell lines. The flow cytometric assay analysis showed that the most active compound arrested the cell cycle at G0/G1 phase and effectively induced apoptosis [32]. By keeping the same interest, a novel 1*H*-pyrazolo[4,3-b]pyridine-3-amine scaffold was synthesized and evaluated in vivo (rodent models) against Parkinson’s disease (PD) which showed remarkable efficacy and selectivity. Adding to this study, a novel derivative originating from the parent compound was synthesized (**1d**), the potency and selectivity was evaluated by positive allosteric modulation (PAM) of metabotropic glutamate receptors (mGlu_4_). The SAR revealed that by using a picolinamide as a core scaffold, a variety of analogs were investigated such as; introduction of indazole, indole and polyazo systems by replacement of phenyl rings, elaboration of *N*-1 and *C*-3 positions by different functional groups and finally, an additional methyl group on the indazole moiety led to the increase in potency against PD (EC_50_ = 43 nM) [33]. As, 1*H*-pyrazolo[4,3-*b*]pyridine derivatives have been reported in literature as protein kinase inhibitors. Therefore, a new series of 3,6-diaryl-1*H*-pyrazolo[4,3-*b*]pyridine derivatives were developed as potential drug targets for anaplastic lymphoma kinase (ALK). It was concluded on the basis of detailed SAR studies that the hydroxyphenyl substitution on 6-position of 1*H*-pyrazolo[4,3-*b*]pyridine was a crucial factor in inhibiting ALK enzymatic activity (IC_50_ 1.58 nM) with the *ortho* substitution of fluorine atom (**1e**). The promising activity was observed due to the hydrogen bonding interactions of hydroxyl group of phenol with residues Lys1150 and Asp1270 which were also seen in other ALK known drugs [34] (Figure 1).

Imidazo pyridine derivatives have also been studied widely and were found probable pharmacological agents in the domains of cardiology, neurology, endocrinology and oncology. Among them, Imidazo[1,2-*a*]pyridine have exhibited many potential therapeutic applications such as; anxiolytic, insomnia, motility stimulant and for the treatment of osteoporosis. In this interest, a novel series of 2-(3,4-dimethoxyphenyl)-6-(1,2,3,6-tetrahydropyridin-4-yl)imidazo[1,2-*a*]pyridine analogues (**2a**) were evaluated in vitro for antiproliferative activity against a panel of cancer cell lines viz., A549 (lung cancer), HeLa (cervical cancer), B16F10 (melanoma) and found to show potent anticancer potential with IC_50_ range 2.0–20.0 µM [35]. Similarly, a new series of imidazo[4,5-*b*]pyridines with 5,6-dichloro substitution and 2-alkylamino-methyl or ethyl groups along with their respective *N^1^* or *N^2^*-tosylates was prepared. The anti-HBV activity of the synthesized compounds was evaluated in efficient infection system (in vitro) with dose-dependent curve analysis. The EC_50_ and CC_50_ were determined, and the SAR studies were established for the most active compound. In the light of these experiments, it was concluded that the highest antiviral activity was attributed to the monochloro diethylamino ethyl substituted derivative with EC_50_ = 13.53 ± 0.92 (**2b**), as it intervened the final stages of viral DNA replication or nucleocapsid maturation, comparable to interferon [36].

Based on the therapeutic chemistry of thiazolidinone derivatives, a new series of 2-imino-3-aryl-4-thiazolidinone derivatives containing pyridine scaffold (**2c**) as an effective compound, was synthesized and evaluated against some *Alternaria* and *Aspergillus* fungal strains. The detailed SAR studies have shown that the incorporation of phenyl and hetaryl ring, substitution with electron withdrawing groups such as chloro, fluro and bromo, addition of hydroxyl group at the phenyl ring and finally the incorporation of arylazo groups to the basic structural framework of thiazolidinone derivatives have increased the efficacy (80–85%) of the synthesized compounds against tested fungal strains [37].

Recently, a series of novel thieno[2,3-*b*]pyridine derivatives were designed and screened via MTT assay for cytotoxic activities against normal fibroblasts and four cancer cell lines (HepG-2, Caco-2, MCF-7 and PC-3). The study has suggested that potential inhibitors of cancer targets such as protein kinases and epidermal growth factor receptors have structural features such as; heterocyclic aromatic ring as a core, hydrogen bond donor/acceptor pair (amide or urea moiety), a terminal aromatic ring with sufficient space of various substitutions. In the light of these studies, it was concluded by kinase inhibitory assays and flow cytometric data analyses that the assembly of thienopyridine core, an amide linker and *p*-tolyl group (**2d**) conferred the highest kinase inhibition (PIM-1), best epidermal growth factor receptor (VEGFR) suppression and caspase 3/7 activation compared to the respective standard drugs [38].

**Figure 2:**
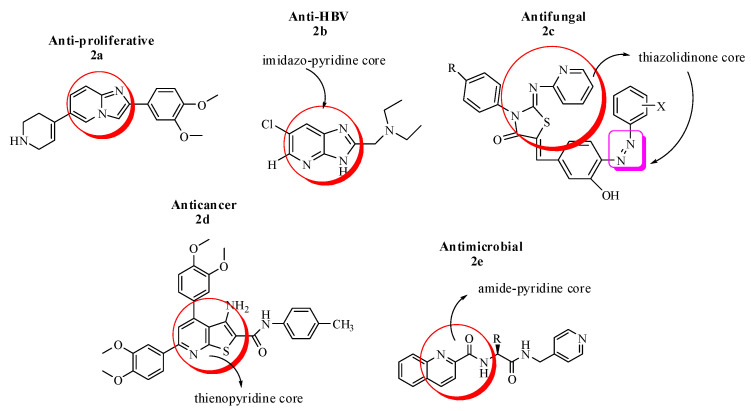
Most active imidazo, thiazo, thieno and amide–pyridine analogues.

A novel series of compounds with amide-pyridine scaffold (**2e**) was designed to treat the increasing incidence of drug-resistant fungal strains by inhibiting the target active sites of squalene cyclooxygenase (SE) and 14α-demethylase (CYP51) pharmacophores. The in vitro antifungal evaluation suggested that the title compounds have broad-spectrum potential against drug-resistant fungal strains with MIC range of 0.125–2.00 µg/mL compared to the standard drug fluconazole. In addition, the ADME and toxicity assays were also performed which further demonstrated that amide-pyridine derivatives did not show genotoxicity and have drug-like properties [39] (Figure 2).

## 2. Pharmacological Properties of Phenol Derivatives

**Figure 3:**
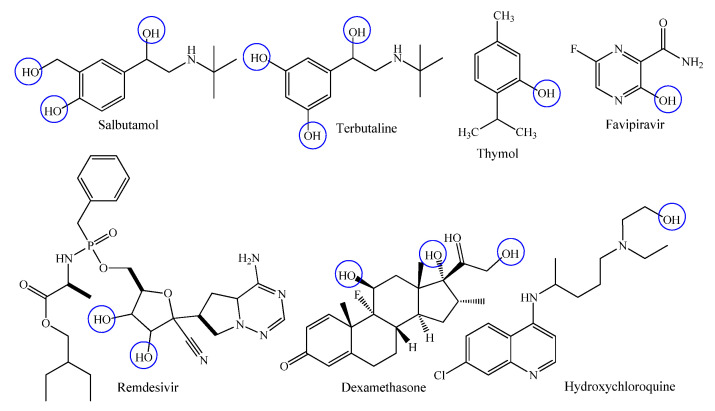
Chemical structures of commercially available phenolic drugs.

Phenolic compounds are simple and naturally occurring organic compounds bearing an aromatic ring with one or more hydroxyl groups (with or without side chain), they received considerable attention due to their biological functions such as anti-carcinogen, anti-ageing, anti-diabetic, anti-mutagen and immunostimulatory roles [40,41]. Among the most extensively administered phenolic drugs, salbutamol, terbutaline, thymol, favipiravir, remdesivir, dexamethasone and hydrochloroquine are highly recognized β_2_-adrenergic receptor agonists, antiseptic, antimicrobial agent and antiviral agents, respectively (Figure 3) [42,43].

In this interest, a new series of phenolic cinnamic acid derivatives was designed with the aim to selectively inhibit cyclooxygenase (COX-1 and COX-2) enzymes. The results of SAR studies revealed that the presence of phenolic hydroxyl groups was an essential factor in the inhibition of inflammatory enzyme (COX). Hence, it was concluded form the initial screening data that most active compounds (**4a**–**d**) could possibly be considered as starting point hit compounds (IC_50_ range 1.09 ± 0.09 to 3.00 ± 0.3 µM) for further optimization as anti-inflammatory non-steroidal drugs [44]. A novel series of phenolic *N*-monosubstituted carbamates as new anti-mycobacterial drug targets was designed against broad-spectrum mycobacterial strains (*Mycobacterium tuberculosis* H_37_Ra, H_37_Rv including multidrug and extensively drug-resistant strains, *Mycobacterium avium*, *Mycobacterium kansasii*, *Mycobacterium aurum* and *Mycobacterium smegmatis*). The title compounds were also evaluated for cytotoxic potential on HepG cells, in which all compounds were found non-toxic. The minimum inhibitory concentration (MIC) was found in the range of 0.79–0.50 µM comparable to the standard drug isoniazid. Hence, it was concluded after detailed SAR studies that the presence of Adamantan-1-yl group was the most efficient moiety of phenyl carbamate (**4e**) for a broad-spectrum antimycobacterial potential [45].

**Figure 4:**
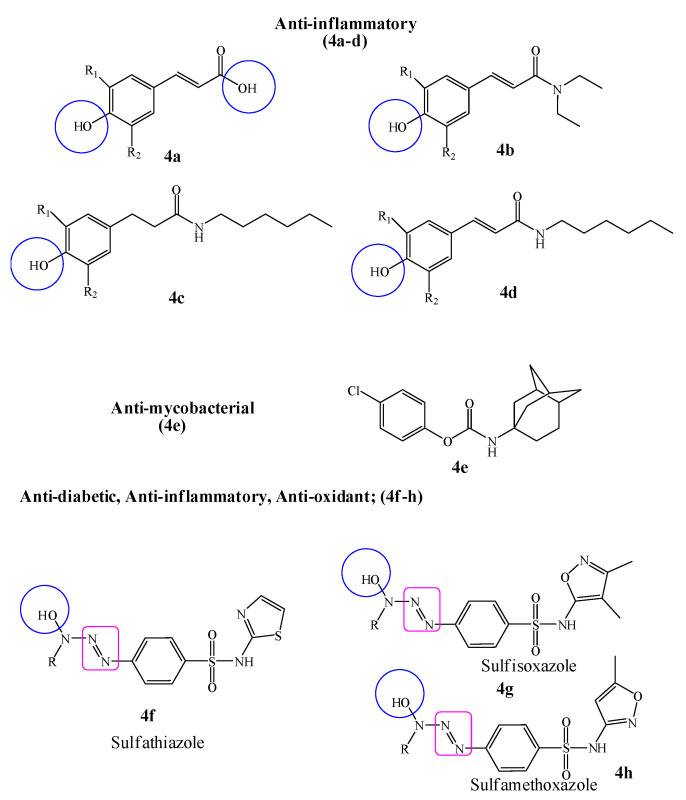
Biologically active phenolic cinnamic acid, phenolic carbamate and sulfa drugs based hydroxytriazenes derivatives; R, R_1_ and R_2_ are alkyl, alcoholic, alkoxy and *tert*–butyl moieties.

Hydroxytriazenes are a class of compounds containing α-hydroxyl group relative to diazo group, have versatile pharmacological properties including lipid lowering, antidiabetic, antioxidant, anti-inflammatory, antimicrobial and analgesic agents. In view of this, an attempt has been made to study the anti-diabetic, anti-inflammatory and antioxidant effects of sulfa drugs-based hydroxytriazenes (**4f**–**h**). It was suggested that the compounds showed significant α-amylase and α-glucosidase inhibition potential with IC_50_ values ranging from 122 to 341 µg/mL. The promising anti-inflammatory (89% after 4 h of treatment) and radical scavenging potential (IC_50_ 54.12 µg/mL) highlighted the multifunctional role of hydroxytriazenes. It was also concluded that the polar functionalities such as –OH around the heterocyclic rings of triazene compounds enhanced the inhibition potential against glucosidase and amylase [46] (Figure 4).

A series of new phenyl acrylamide derivatives were designed as non-nucleoside anti-HBV target leads (**5a**). Among the tested derivatives, some were found to be potentially active in inhibiting HBV DNA replication with IC_50_ 0.19 µM. The structure activity studies further elucidated that the target lead derivative bound the dimer-dimer interface of the HBV protein core with low toxicity and diverse mechanism of action due to the presence of *para*-substituents of –Br, –F and –Cl groups, respectively [47].

**Figure 5:**
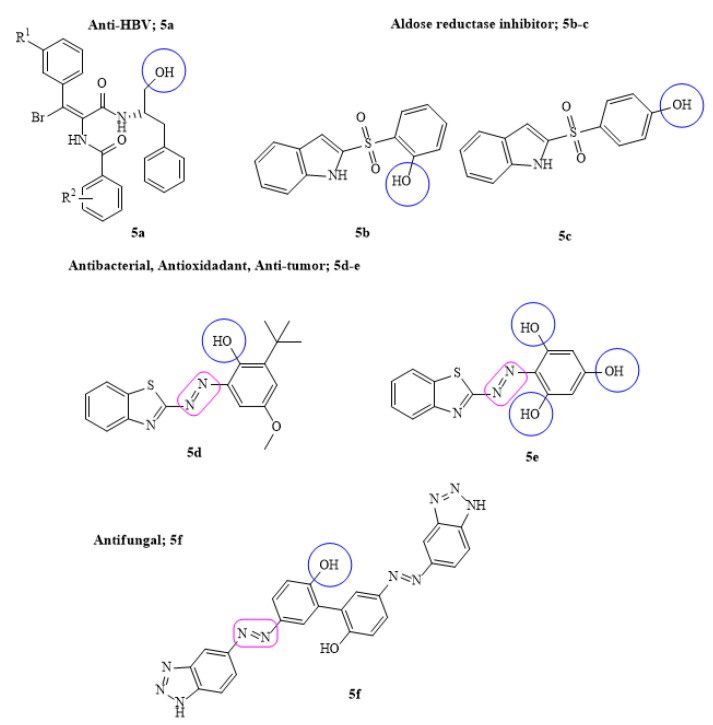
Some of the most active phenyl acrylamide, sulfonyl phenol and azo–phenol derivatives; R^1^ represent alkyl, alkoxy and halo substituent.

The key approach to major pathological conditions such as neuropathy, angiopathy, chronic hyperglycemia and inflammation is to target aldose reductase enzyme. Several carboxylic acid derivatives are proven to be the inhibitors of aldose reductase (ALR2) but due to their poor cell permeability, focus on alternate inhibitors has been greatly enhanced. In this regard, some new sulfonyl-phenol derivatives by replacing carboxylic acid moiety having equipotency and improved permeability were designed. It was suggested that the presence of *p*-sulfonyl-phenol (**5b**–**c**) group on the indole ring was suitably positioned towards the anionic pocket of ALR2 which made all the necessary interactions to be considered in future for lead optimization [48]. Similarly, a series of new benzotriazole-azo-phenol derivatives were prepared and screened against six phytopathogenic fungal strains, i.e., *F. graminearum*, *F. solani*, *A. alternate*, *V. mali*, *B. cinerea* and *C. lunata*. The SAR and broad-spectrum antifungal screening data suggested that some of the synthesized derivatives (**5f**) showed 3.5–10-fold therapeutic and protective effects against tested fungal strains compared to the standard drug carbendazim. It was noteworthy that the presence of biphenol (dihydroxy) groups on phenyl rings exhibited better inhibition of fungal strains [49]. Keeping the same interest, a new series of bioactive azo derivatives fused with benzothiazole coupled with phenolic molecules such as phlorogucinal, 2,4-di-*tert*-butyl and 2,6-di-*tert*-butyl phenol was designed. The versatile biological applications such as antibacterial, antioxidant, anti-tumor and EGFR protein were assessed. The obtained data were compared with the respective standard drugs and suggested that few of the designed drugs (**5d**–**e**) were potentially active against free radicals, pathogenic bacterial strains and showed interactions with EFGR protein due to the presence of phenolic ring fused with benzothiazole through diazo group that led to the increase in electron density and more potency [50] (Figure 5).

## 3. Therapeutic Potential of Azo Moiety

### 3.1. Syntheses Routes of Azo Derivatives

**Scheme 1:**
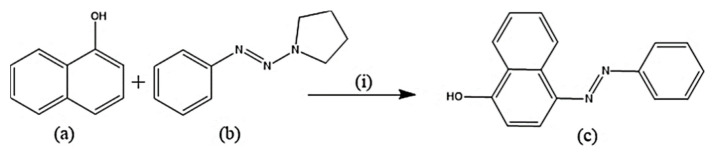
Synthesis of azodyes (**c**). (i) (**a**) (0.2 mmol), (**b**) (0.2 mmol), Ionic liquids (0.2 mmol) [51], H_2_O (1 mmol), room temperature, 4 hours.

**Scheme 2:**
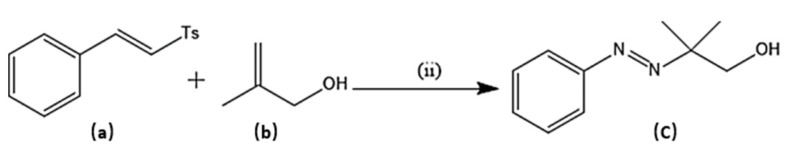
Synthesis of target molecule (**c**); (ii) (**a**) (0.2 mmol), (**b**) (0.4 mmol), PhSiH_3_ (2 eq), metal salts (20 mol %), additive (1 eq), methanol (2 ml), room temperature.

**Scheme 3:**
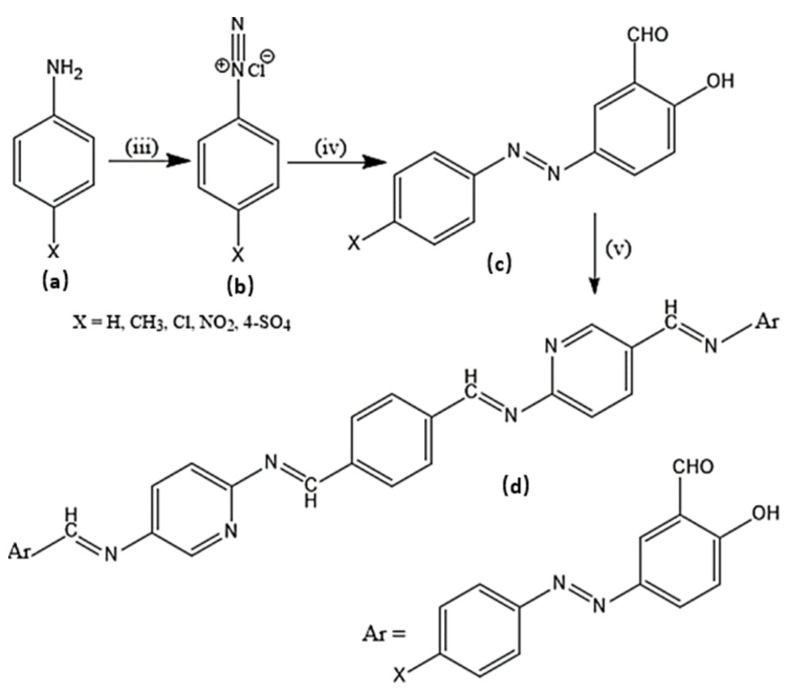
Synthesis of target molecule (**d**); (iii) Sodium nitrite, HCl (iv) Salicylaldehyde (v) Coupling with amine derivatives.

**Scheme 4:**
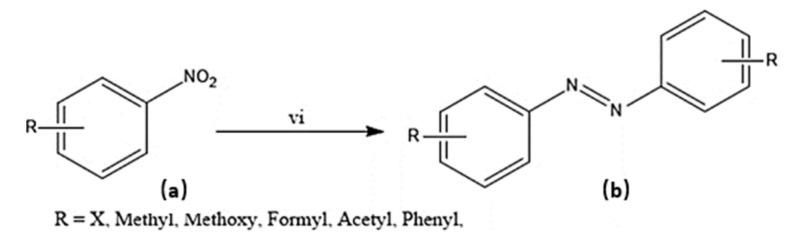
Synthesis of target molecule (**b**); (vi) (**a**) (1 eq under nitrogen atmosphere), NaOH (0.2 eq), Au@OC_1_^R^ (0.5 mol % Au), 2-propanol (15 mL), 2 hours, 30 °C.

Several experimental procedures have been employed for the syntheses of azo compounds; Recently, a facile and eco-friendly procedure for azo dyes synthesis was developed by using Bronsted acidic ionic liquids (ILs) as a reaction promoter with aryltriazenes as coupling agents, water was used as solvent at room temperature under air and metal-free environment (Scheme 1). They have observed excellent product yields (>90%), recyclability of solvents and advantages of the up-scaled application [51]. Similarly, in another novel synthesis attempt was made by implying Fe(II)-catalyzed dehydrogenative coupling of aryl diazo sulfone for producing alkyl arylazo derivatives under mild reaction conditions (Scheme 2) and yielded diverse azo compounds which would be otherwise not feasible using other approaches [52]. Another novel method was conducted for the synthesis of azo-Schiff bases by microwave irradiation route (200 W for 10 min) due to its advantages such as robustness, cleanliness and economical approach compared to the conventional methods of synthesis (Scheme 3). The produced azo-Schiff derivatives were found to be more photostable [53]. Another novel strategy was adopted for eco-friendly, upscaled and one-step conversion of nitroarenes to azo compounds via the photocatalytic route implying gold nanoparticles (Scheme 4) was found to have additional advantages of high selectivity, chemical stability, reproducibility and excellent product yield in less time [54].

**Scheme 5:**
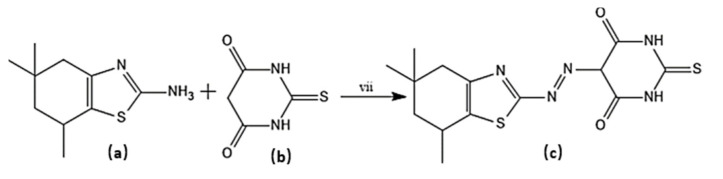
Synthesis of target molecule (**c**); (vii) NaNO_2_/HCl, 0-5 °C, Coupling of (**a**) with (**b**) at pH 5–6.

A lot of work has been conducted on the traditional approach of diazo coupling reaction for azo dyes synthesis. In this regard, reported novel series of azo disperse dyes via diazo-coupling reaction at 0–5 °C between 5,5,7-trimethyl-4,5,6,7-tetrahydro-1, 3-benzothiazol-2-amine and 2-thioxodihydropyrimidine-4,6(1*H*,5*H*)-dione (Scheme 5). The facile approach resulted in excellent yields [55]. Another example of diazo-coupling reaction method to synthesize new series of benzothiazole with substituted pyridone/pyrazole as coupling agent is reported [56]. Briefly, substituted aromatic amine was mixed with conc. HCl/H_2_SO_4_ and cooled to 0–5 °C. To this reaction mixture, sodium nitrite was added drop-wise with constant stirring for 1–1.5 h. The resulting diazonium salt solution was immediately mixed with the coupling agent to produce azo dyes.

The solvent-free conditions are being used in recent times to overcome the researcher’s concerns such as green chemistry, cost-effectiveness, easiness, reaction time and mildness, etc. By keeping these concerns in mind, a new and convenient method was reported for the synthesis of 5-[(*E)*]-(5-sulfanyl-1,3,4-thiadiazol-2-yl)diazenyl]-6-thioxo-dihydropyrimidine-2,4(1*H*,3*H*)dione by the diazo coupling of 5-amino-1,3,4 thiadiazole-2-thiol with thio-barbituric acid under solvent free conditions without using any catalyst. The comparison was made in terms of duration of reaction and product yield with the conventional approaches, it was concluded that the solvent-free approach was advantageous in terms of cost-effectiveness, simplicity of experimental design and recovery of product [57]. A series of diazenyl Schiff bases was synthesized by reacting salicylaldehyde incorporating azo derivatives with substituted aromatic amines in the presence of acetic acid as a catalyst. It was found that the use of catalyst decreased the reaction time and enhanced the efficiency of the reaction [58].

### 3.2. Azo as Therapeutic Agents

**Figure 6:**
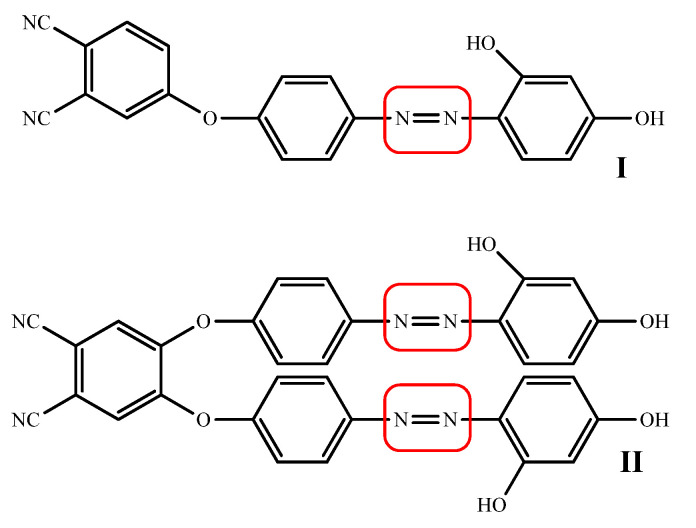
Biologically active parent compounds of phthalocyanine incorporated azo moiety.

Phthalocyanines are diverse chromophores with interesting physical, chemical and optical properties and are reported widely with different substituents. However, phthalocyanines bearing azo pharmacophore are less discovered and discussed. The designing of new metallo-phthalocyanine incorporated azo derivatives and their biological potential such as anticancer, antibiotic and antioxidant were evaluated and compared with the standard drugs, respectively. The screening data suggested that the metal complexes of parent compounds were highly active in radical scavenging activity (~70% inhibition compared to the parent compounds ~35% inhibition). However, the antimicrobial and cytotoxicity potential was observed in parent structures (Figure 6) [59].

**Figure 7:**
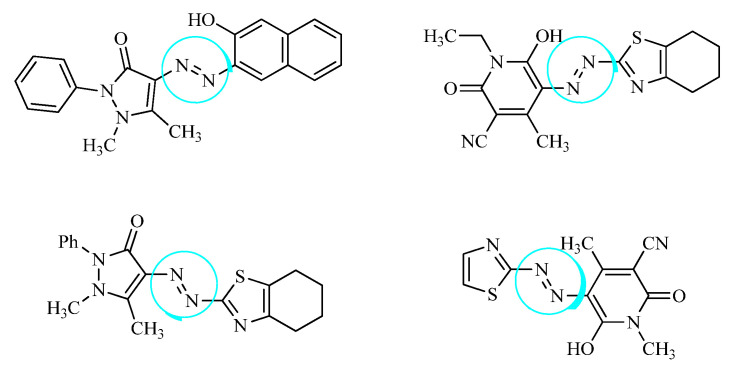
Most effective azo derivatives with DNA binding properties.

The azo moiety bearing derivatives also exhibit DNA cleavage activity primarily due to the existence of nitrogen and oxygen atoms on the aromatic rings with the capability to stack the bases of DNA. an azo derivative from 4-aminoantipyrine and found effective in DNA cleaving activity by using pUC18 [60], the DNA binding activities of tetrahydro-1,3-benzothiazole incorporated azo dyes was found to have effective intercalation properties with circulating tumor DNA (CT-DNA) [61]. Another considerable DNA cleavage efficiency of 2-aminothiazole incorporated azo dyes against supercoiled pBR322 DNA is also reported in literature [62]. All efficient DNA cleaving azo derivatives are mentioned in (Figure 7).

**Figure 8:**
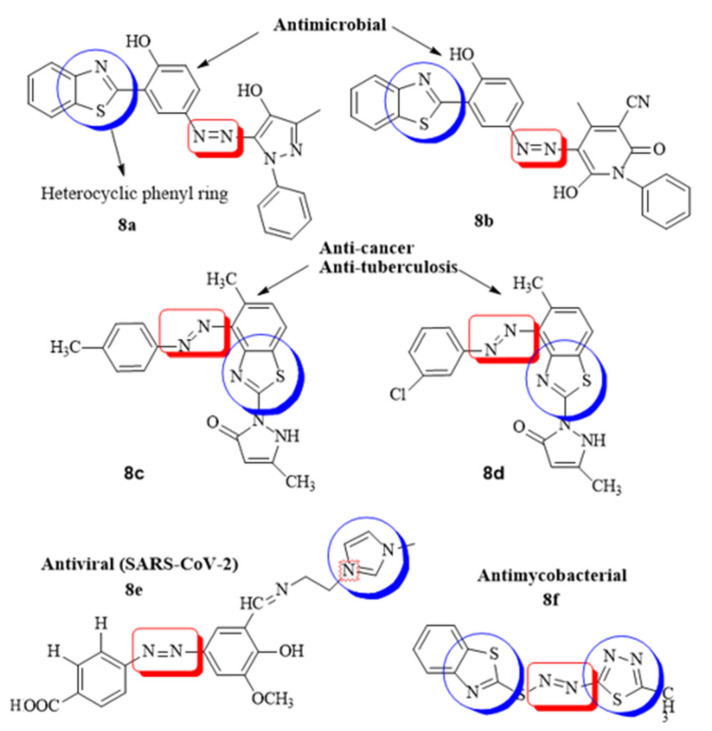
Some of the reported heterocyclic azo derivatives.

Heterocyclic azo dyes, containing sulphur and nitrogen in the phenyl ring, can be easily produced through diazo coupling reaction and comprise an important class of aromatic compounds in medicinal chemistry. The in vitro/vivo biological properties of heterocyclic azo derivatives such as antimycobacterial, antimicrobial, anti-tumor, anthelmintic, etc., has been studied widely [63]. A series of azo clubbed benzothiazole derivatives and their antibacterial potential against *S. aureus* and *E. coli* was studied in detail. It was concluded after the docking simulation that the most active azo clubbed compounds (**8a,b**) could be optimized into lead molecules for most potent antimicrobial agents compared to the standard ciprofloxacin [56]. A novel series of benzothiazole disperse azo dyes was evaluated for anticancer and anti-tuberculosis activity, the results suggested moderate anti-proliferative potential of all the compounds while –CH_3_ and –Cl substituted heterocyclic azo derivatives (**8c,d**) exhibited highest anti-mycobacterial efficacy as compared to the standard drug pyrazinamide [61]. A novel imidazole anchored azo derivatives against the main protease (6LU7) of novel corona virus (COVID-19) was analyzed and it was revealed that the meta-substituted –COOH azo imidazole derivative (**8e**) showed significant affinity with the main protease of SARS-CoV-2 with binding affinity −8.1 kcal/mol. The overall protein–ligand interaction studies showed that the novel compounds could act as the potential drug candidate against COVID-19 [64]. In the similar interest, a new series of sulfur-containing heterocyclic azo compounds with appreciable antibacterial and antimycobacterial efficacy was designed. The most active derivative (**8f**) was found to have significant interaction properties with the target receptor of mycobacterium RpsA (−5.5 kcal/mol) due to the number of heteroatoms in the phenyl ring [65] (Figure 8).

**Figure 9:**
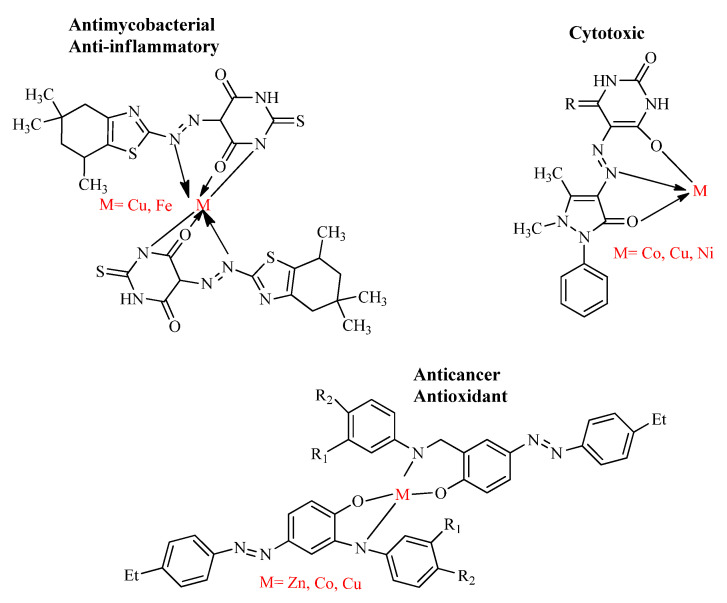
Bioactive metal incorporated azo derivatives.

The investigations on the development of metal-based drugs have been an interesting area for the bioinorganic chemists since the discovery of the effective metal incorporated anticancer drug cisplatin [63]. Therefore, recent studies are focused on the design and synthesis of the nitrogen- and sulfur-containing heterocyclic azo dyes and their metal chelates due to the decreased toxicity, excellent and versatile biological properties, such as its antimalarial, antitubercular, anti-inflammatory, anti-leishmanial and anti-oxidant effects [66,67]. The metal complexes, Cu(II) and Fe(III), of azo dyes derived from benzothiazole are pharmacologically effective against the drug-resistant strain of *M. tuberculosis* (MIC value 1.6 µg/mL) and matrix metallo-protein enzymes (88–95% inhibition) [55]. Similarly, the metal complexes; Zn(II), Co(II) and Cu(II) of azo-azomethine ligands were found to possess more efficient in vitro proliferation inhibitory potential against non-small lung cancer and prostate cancer compared to the parent ligands [68] and the metal salts; Co(II), Cu(II) and Ni(II) of 4-amino antipyrine azo derivatives were screened in vitro for the cytotoxic potential. At the concentration of 100 µg/mL, the metal complexes of Ni(II) and Co(II) showed appreciable cytotoxic effect by decreasing the cell viability to 50% [69] (Figure 9).

### 3.3. Azo Dyes as Chemosensors

**Figure 10:**
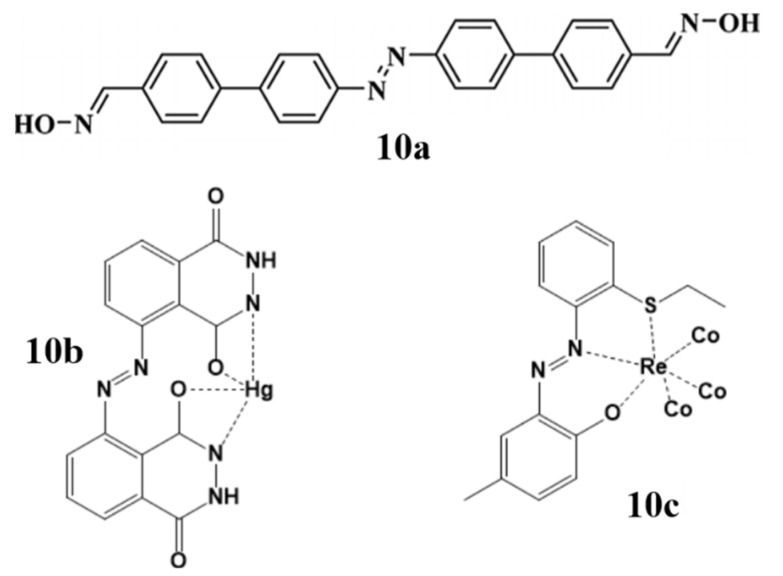
Some reported azo derived chemosensors for metal detection and bio-imaging studies.

The increasing toxic concentrations of heavy metal ions such as lead (Pb), copper (Cu), mercury (Hg), cyanide (CN^−^) and iodide (I^−^) in the environment cause various cardiovascular and neurological problems worldwide [70]. World health organization (WHO) insists on taking precautions to limit the toxic effects of heavy metals around the globe. In this regard, various strategies have been reported for the detection of metal ions, from which fluorescence–based sensors have gained much attention due to their high sensitivity, selectivity, simple protocols and non-destructive nature [71,72,73]. One of the most practiced fluorescence-based sensing is chemo-sensing, which employs a small molecule that signals the presence of an analyte by binding to it and in result the variation in the molecular properties is recorded. A large number of chemosensors have been reported and investigated so far, recently, a versatile biphenyl-azo conjugated aldoxime (**10a**) was found to be a selective probe for the detection of Hg^2+^ and F^−^ over a wide range of metal analytes in intracellular living system [74]. Another selective probe for the detection of Hg^2+^ in the living cells, a C_2_ symmetric azo derivative of luminol (Azolum) (**10b**) that has the non-destructive cellular permeability and could quench the fluorescence only in the presence of Hg^2+^ ions [75]. In a separate experiment, a selective and sensitive biomarker rhenium(I) carbonyl complex bearing azo-phenol ligand (**10c**) for cancer cell bio-imaging was synthesized, the cytotoxicity of this complex was determined and found to be moderately toxic for the cells [76] (Figure 10).

Colorimetric chemosensors have been widely used in the recent years due to the number of advantages such as its low cost, ease of detectability with the naked eye, lower equipment requirement, quick response time and practical applications in the fields of biochemistry, food processing, pharmaceuticals and clinical analysis. Among colorimetric chemosensors, azo chromophores display advantages such as; high sensitivity/selectivity, aqueous solubility, strong photophysical properties, real-time monitoring with naked-eye visibility and excellent coordination ability with transition metal ions [77,78].

**Figure 11:**
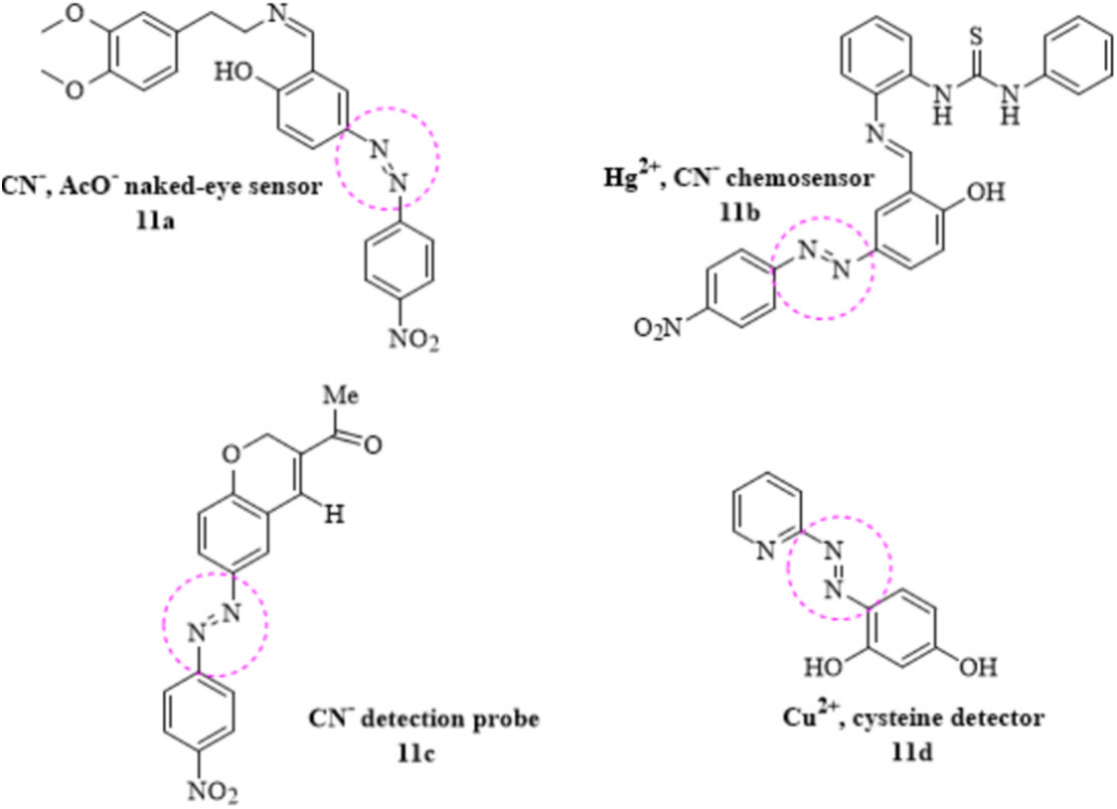
Some reported azo–based chemosensors.

In the same interest of discovering most suitable probe with above-mentioned properties, the chemosensing behavior of a new azo-azomethine phenol (**11a**) derivative was investigated for the selective detection of CN^−^ AcO^−^ and carboxylate anions from sodium diclofenac. The visible change in color shift from orange to dark blue was detected as a result of hydrogen bonding interactions between ligand and anions and it was concluded that the new azo derived chemosensor could be used as the quantitative tool in the detection of sodium diclofenac in the oral pill [79]. In a recent research, a novel azo-benzylidene-thiourea naked-eye chromogenic sensor was synthesized for the qualitative measurement of Hg^2+^ and CN^−^ ions (**11b**) with the detection concentration of 4.89 µM and 2.23 µM, respectively [80]. Similarly, a novel azo dye-based CN^−^ detection probe was developed; the highly selective chemosensor underwent ring opening reaction upon interaction with the CN^−^ ions (**11c**) with a dramatic color change in the aqueous solution [81]. Another sensitive chemosensor pyridyl-azo resorcinol (**11d**) for the sequential detection of Cu^2+^ and cysteine (Cys) in aqueous solution was developed. The detection limit (31 nmol/L) for Cu^2+^ was found to be lowered than reported by WHO. Surprisingly, the subsequent ligand–Cu^2+^ complex was selectively able to probe cysteine residues in the solution with the detection limit ~72 nmol/L. These findings offered a potential way to detect a wide range of analytes [82] (Figure 11).

## 4. Conclusions

Despite the tremendous advancements in the exploration of therapeutic potency of heterocyclic compounds, continuous efforts are still in progress in search of more potent, new and safe synthetic derivatives. Herein, the recent pharmacological perspectives of pyridine scaffolds, phenolic compounds and derivatives of azo moiety have been illustrated. The structure–activity relationship studies of the most potent synthetic derivatives are also discussed.

In the medicinal chemistry of pyridines section, the significance of the addition of the aryl azo group (**2d**) is highlighted, the incorporation of the diazo group in the pyridinyl backbone has remarkably increased (~10 fold) the antifungal potential of the heterocyclic derivative. In pharmacological properties of phenolic compounds section, special focus is given to the hydroxytriazenes due to the presence of diazo group relative to the α-hydroxyl group (**4g**–**h**) that has enhanced the anti-diabetic activity of the synthetic derivative. Similarly, the azo-phenol derivatives (**5d**–**f**) caused an increase in the electron density of the synthetic molecules which ultimately leads to the enhanced antifungal, antibacterial and antioxidant properties, respectively. The literature also highlighted the antiviral efficacy of a new azo-imidazole compound as SARS-CoV-2 inhibitor (**8e**), which particularly highlights the importance of azo moiety to address the current pandemic situation (COVID-19). The reported azo-imidazole derivative can be further optimized structurally to be developed into more efficient anti-COV drug.

In summary, it can be concluded that the incorporation of the azo linker to the pyridine framework and phenolic substituent have synergistically enhanced the therapeutic properties of the synthetic compounds. Alongside the inhibitory properties of the azo derivatives, the chemo/bio sensing behavior is also discussed indicating the bright future for the development of heterocyclic azo-phenol derivatives as therapeutic and diagnostic tool.

## Data Availability

Excluded as study did not report any data.
